# Temporal Variation of the Profile and Concentrations of Paralytic Shellfish Toxins and Tetrodotoxin in the Scallop, *Patinopecten yessoensis*, Cultured in a Bay of East Japan

**DOI:** 10.3390/md17120653

**Published:** 2019-11-21

**Authors:** Satoshi Numano, Yuta Kudo, Yuko Cho, Keiichi Konoki, Mari Yotsu-Yamashita

**Affiliations:** Graduate School of Agricultural Science, Tohoku University, 468-1 Aramaki-Aza-Aoba, Aoba-ku, Sendai 980-8572, Japan; satoshi.numano.s8@dc.tohoku.ac.jp (S.N.); yuta.kudo.d5@tohoku.ac.jp (Y.K.); yuko.cho.a4@tohoku.ac.jp (Y.C.); keiichi.konoki.b2@tohoku.ac.jp (K.K.)

**Keywords:** scallop, paralytic shellfish toxins, tetrodotoxin, HILIC-MS/MS, *Alexandrium tamarense*

## Abstract

Paralytic shellfish toxins (PSTs) are the major neurotoxic contaminants of edible bivalves in Japan. Tetrodotoxin (TTX) was recently detected in bivalve shellfish around the world, drawing widespread attention. In Japan, high levels of TTX were reported in the digestive gland of the scallop, *Patinopecten yessoensis*, in 1993; however, no new data have emerged since then. In this study, we simultaneously analyzed PSTs and TTX in scallops cultured in a bay of east Japan using hydrophilic interaction chromatography (HILIC)-MS/MS. These scallops were temporally collected from April to December 2017. The highest concentration of PSTs (182 µmol/kg, total congeners) in the hepatopancreas was detected in samples collected on May 23, lined to the cell density of the dinoflagellate, *Alexandrium tamarense*, in seawater around the scallops, whereas the highest concentration of TTX (421 nmol/kg) was detected in samples collected on August 22. Contrary to the previous report, temporal variation of the PSTs and TTX concentrations did not coincide. The highest concentration of TTX in the entire edible tissues was 7.3 µg/kg (23 nmol/kg) in samples obtained on August 22, which was lower than the European Food Safety Authority (EFSA)-proposed threshold, 44 µg TTX equivalents/kg shellfish meat. In addition, 12β-deoxygonyautoxin 3 was firstly identified in scallops.

## 1. Introduction

**Figure 1:**
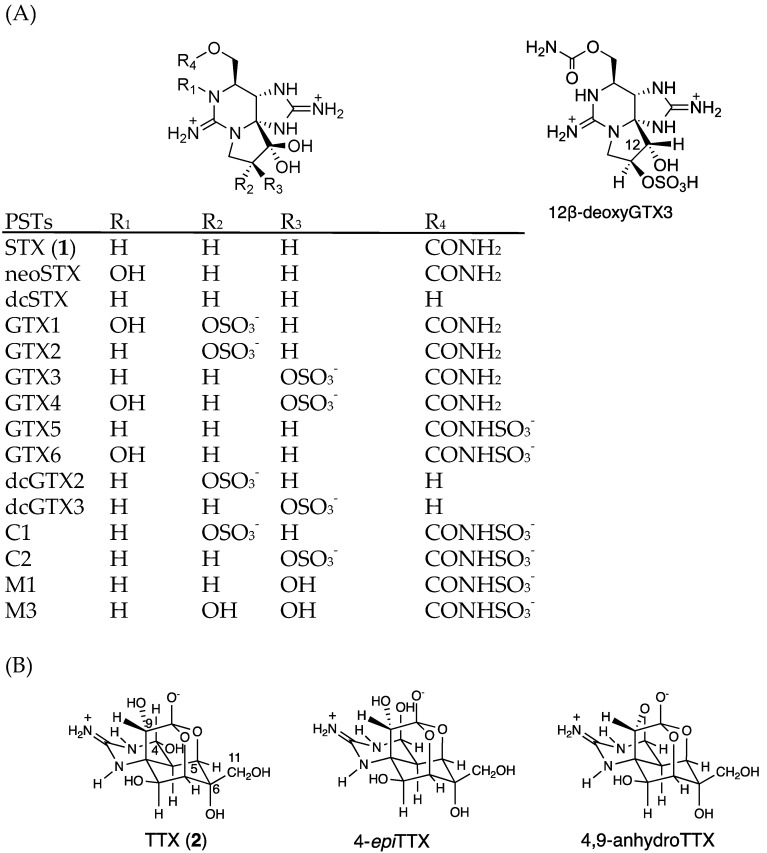
The structures of major PSTs (**A**) and TTXs (**B**). (GTX; gonyautoxin, dc-; decarbamoyl).

Saxitoxin (STX, **1**, Figure 1A) [1,2] and approximately 60 natural analogues of STX are commonly known as paralytic shellfish toxins (PSTs) [3]. These toxins are potent and specific voltage-gated sodium channel (Na_v_) blockers, present in nerve and muscle cells [4]. PSTs are produced by dinoflagellates in marine environments and by cyanobacteria [5,6] in terrestrial environments. In marine environments, PSTs accumulate in some species of shellfish, including economically important seafood species such as scallops, oysters, and mussels. PSTs in such shellfish are globally regulated. Codex [7], the European Union (EU) [8], and the United States of America (USA) [9] have determined the upper limit of PSTs as 800 μg of STX equivalents/kg (2673 nmol/kg) shellfish meat.

Tetrodotoxin (TTX, **2**, Figure 1B) is also a specific and potent Na_v_ blocker that shares the same binding site as that of STX [10,11]. Although TTX is known as the pufferfish toxin [12], which occasionally causes lethal food poisoning, this toxin has been found in a wide-range of marine and terrestrial animals such as octopuses [13], crabs [14], starfish [15], newts [16], and frogs [17]. In some species of pufferfish and crabs, both TTX and STX were detected [18,19]. Many TTX-producing marine bacteria have been reported, such as *Vibrio, Bacillus, Aeromonas, Alteromonas,* and *Pseudomonas* [20,21,22]. However, the biosynthetic genes of TTX have not yet been reported. We are approaching this problem by identifying natural analogues of TTX and developing analytical methods for them [23,24,25,26,27,28,29].

In 2014, TTX was detected in the surf clam, *Paphies australis,* in New Zealand [30]. After that, this toxin was frequently detected in the common blue mussel, *Mytilus edulis,* and in the Pacific oyster, *Crassostrea gigas,* in 2015 [31], and in the oysters, *Ostrea edulis*, mussels, and the hard clam, *Mercenaria mercenaria*, in 2017 [32] in England. Continuously, it was detected in mussels in Greece in 2012 and 2015 [33], in mussels and oysters in the Netherlands in 2016 and 2018 [34] and in Italy in 2018 [35], and in *Ruditapes philippinarum*, *Sinonovacula constricta*, *M. edulis,* and *M. coruscus* in China in 2013–2014 [36]. According to a recent review, TTX has been reported in 11 species of bivalves from seven countries and many species of edible gastropods from five countries [37]. A relationship between TTX concentration, in mussels in Greece, and the prevalence of the dinoflagellate *Prorocentrum minutum* was also proposed [38]. In such a situation, the European Food Safety Authority (EFSA) proposed that a concentration below 44 μg TTX equivalents/kg (138 nmol/kg) shellfish meat was considered not to cause adverse effects in humans [39]. This report further accelerated TTX investigation around the world. After this paper, more papers concerning TTX in Portugal and Spain were published [40,41,42,43].

In Japan, Kodama et al. detected high levels of TTX in 1993 (40 mouse units/g, approximately equivalent to 8 mg/kg, 25 µmol/kg) in the digestive gland of the scallop, *Patinopecten yessoensis,* which was highly contaminated with PSTs (3427 mouse unit/g, approximately equivalents to 685 mg/kg STX, 2289 µmol/kg), using fluorescent HPLC systems for PSTs and TTX individually [44,45]. They reported that the concentrations of both PSTs and TTX peaked on April 26, when the cell density of the dinoflagellate, *Alexandrium tamarense*, in seawater around the scallops rose to its highest. Based on the results, they proposed that *A. tamarense* was the source of TTX in scallops [44,45]. Since then, TTX in Japanese bivalve shellfish has not been reported, although some gastropods have been reported to contain high levels of TTX and cause food poisoning [46].

In this study, our main aims were to: (1) examine the current TTX levels in scallops from a bay of east Japan and (2) compare the temporal variation of the profile and concentrations of PSTs and TTX in scallops to obtain better clues to elucidation of the origin of TTX in marine environments. To achieve them, we firstly set conditions for hydrophilic interaction chromatography (HILIC)-MS/MS to analyze PSTs and TTX simultaneously. Second, we analyzed these toxins in scallops that were cultured in a bay of east Japan and chronologically harvested from one geographic location, from April to December in 2017. In addition, the cell densities of *Alexandrium tamarense* and *Alexandrium catenella* were monitored in the area where the scallops were collected.

## 2. Results

### 2.1. Optimization of HILIC-MS/MS Conditions for Simultaneous Analysis of PSTs and TTX

**Figure 2:**
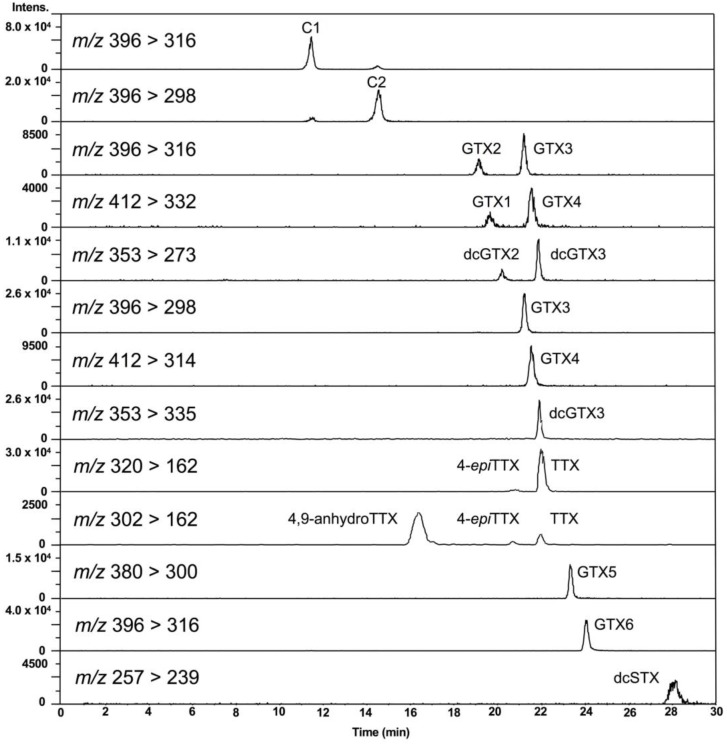
LC-MS/MS chromatograms of PSTs and TTXs standards. The concentration of the standards: C1 879 nmol/L, C2 263 nmol/L, GTX2 1664 nmol/L, GTX3 632 nmol/L, GTX1 1932 nmol/L, GTX4 668 nmol/L, dcGTX2 965 nmol/L, dcGTX3 284 nmol/L, TTX 313 nmol/L, GTX5 132 nmol/L, GTX6 589 nmol/L, dcSTX 759 nmol/L. Injection volume was 5 µL.

First, the conditions for hydrophilic interaction chromatography (HILIC)-MS/MS in multiple reaction monitoring (MRM) mode, especially for MS conditions, were optimized for the simultaneous analysis of PSTs and TTXs based on the previously reported methods [47,48,49,50,51,52]. Liquid chromatography (LC) conditions reported by Thomas et al. [52] were slightly modified for use in this study (see, Section 4.7). Eleven major PSTs (C1, C2, gonyautoxin (GTX) 1-6, dcSTX, dcGTX2-3) along with TTXs (TTX, 4-*epi*TTX, and 4,9-anhydroTTX) (Figure 1) were analyzed. LC-MS/MS chromatograms of the PSTs and TTX standards in the present conditions are shown in Figure 2. The optimized MRM condition, limit of detection (LOD), and limit of quantitation (LOQ) for standard toxins and scallop hepatopancreas extracts (samples) are summarized in Table 1. This HILIC-MS/MS method was validated (Section 4.2).

### 2.2. Analysis of PSTs and TTX in Scallops Using HILIC-MS/MS

**Figure 3:**
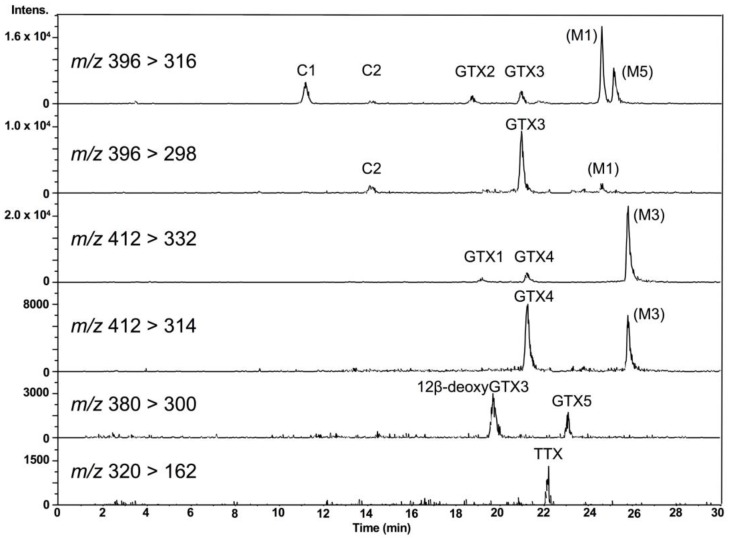
LC-MS/MS chromatograms for PSTs and TTX in the hepatopancreas of scallops harvested on August 1, 2017, using HILIC-MS/MS. 12β-deoxyGTX3 [53] was identified by HR (high resolution)-MS, HR-LC-MS and HR-LC-MS/MS by comparison with synthetic standard (Figures S1 and S2). The presence of M1, M3 and M5 toxins were also suggested, although they were not identified due to lack of standards.

LC-MS/MS chromatograms of PSTs and TTX in the hepatopancreas of scallop samples collected from a bay of east Japan on August 1, 2017 are shown in Figure 3. PSTs (C1, C2, GTX1-5 and dcSTX) and TTX were detected in almost all samples tested, whereas C2, GTX6, dcGTX2-3 and dcSTX were below the LOD (*s*/*n* = 5, Table 1) in all samples. GTX5 was detected in almost all samples, but they were all lower than the LOQ (*s*/*n* = 10). Concentrations of C1, GTX1, GTX2, GTX3, GTX4 and TTX in this sample were determined to be 1645, 48,614, 35,357, 8751, 14,195 and 229 nmol/kg, respectively, using the standard curves drawn for the authentic compounds. The peak detected at 19.7 min at *m*/*z* 380 > 300 was assigned as 12β-deoxygonyautoxin 3 (12β-deoxyGTX3) (Figure 1A) based on HR-MS (*m*/*z* calculated for C_10_H_18_N_7_O_7_ S: 380.0980 [M+H]^+^, found: 380.0989, Δ 2.3 ppm, Figure S1) along with HR-LC-MS and HR-LC-MS/MS by comparison with synthetic 12β-deoxyGTX3 standard [53] (Figures S1 and S2). This compound was further identified using the column switching HR-LC-MS [54] (Figure S3). 12β-deoxyGTX3 was recently identified in the toxic cyanobacterium *Anabaena circinalis* (TA04) by us [53]. This is the first identification of 12-deoxy analogue of PST in bivalves to our best knowledge. In addition to these PSTs, peaks possibly corresponding to the M series toxins (M1, M3 and M5) (Figure 1 for M1 and M3) [55,56,57] were detected at *m*/*z* 396 > 316, *m*/*z* 396 > 298, *m*/*z* 412 > 332, and *m*/*z* 412 > 314, although they were not assigned due to lack of standards. STX and neoSTX like compounds were detected at lower levels than major PSTs. However, they were not qualified and quantified because use of the STX standard, which is provided as a mixture with neoSTX, is strictly controlled by Japan’s governmental regulation body [58].

### 2.3. Identification of TTX in Scallops Using HR-LC-MS and HR-LC-MS/MS

**Figure 4:**
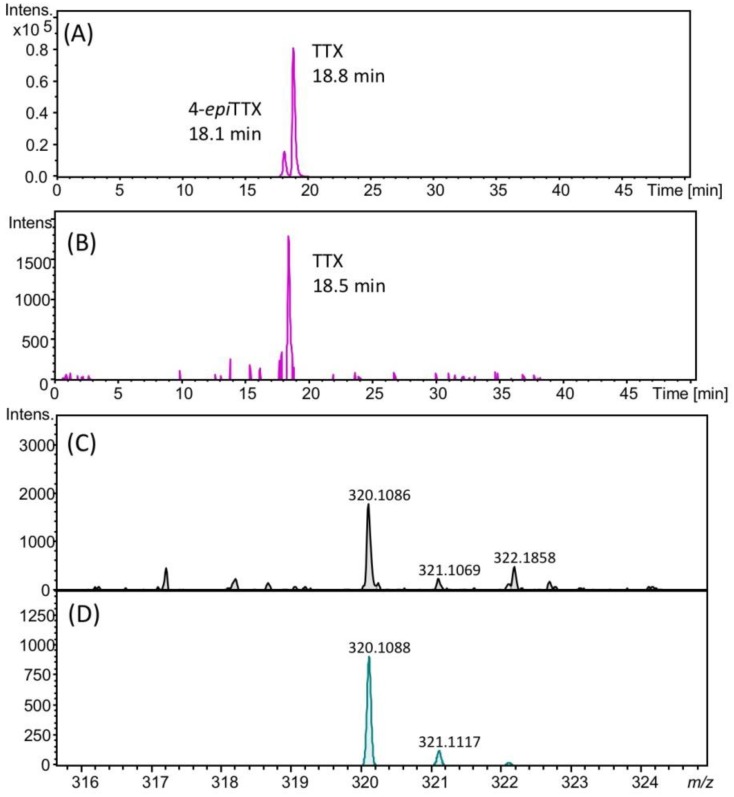
HR-LC-MS chromatograms of authentic TTX (the extracted ion chromatogram (EIC) *m*/*z* 320.1088 ± 0.02) (**A**), TTX in scallops collected on August 22, 2017 (EIC *m*/*z* 320.1088 ± 0.02) (**B**), HR-MS spectrum of TTX in these scallops (**C**), and the theoretical MS spectrum for TTX ([M+H]^+^ C_11_H_18_N_3_O_8_, *m*/*z* 320.1088) (**D**). The retention time of TTX was different from those in Figure 2, Figure 3 and Figure 5, using the slightly different solvent for LC-MS from that for those figures (see, Section 4.8).

**Figure 5:**
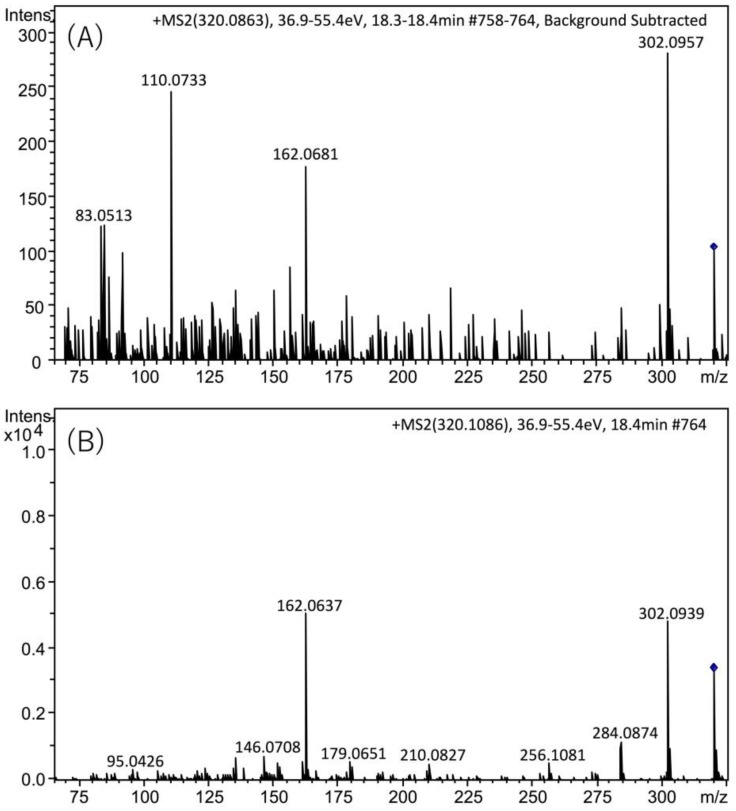
HR-LC-MS/MS spectra of TTX detected in the hepatopancreas of scallops from August 22, 2017 sample (**A**), and authentic standard (**B**). The targeted masses of TTX [M + H]^+^ were *m*/*z* 320.0863 (**A**) and *m*/*z* 320.1086 (**B**). The retention time of TTX was different from that in Figure 2, Figure 3 and Figure 5 in text, using the slightly different solvent for LC-MS from that for those figures (see, Section 4.8).

The presence of TTX in the hepatopancreas in the scallops collected on August 22, 2017 was further confirmed using high resolution (HR)-LC-MS and HR-LC-MS/MS. The HR-LC-MS of the authentic TTX (A) and the sample (B), and HR-MS of TTX in the sample (C) are shown in Figure 4. In the extracted ion chromatogram (EIC) of the sample at m/z 320.1088 ± 0.01, the peak was detected at 18.5 min (Figure 4B), close to the retention of the authentic TTX (18.8 min, Figure 4A). The HR-MS (*m*/*z* calculated for C_11_H_18_N_3_O_8_ : 320.1088 [M+H]^+^, found: 320.1086, Δ 1.3 ppm, Figure 4C) for this peak supported the identification of TTX. The isotope pattern of this ion detected at m/z 320.1086 agreed with the theoretical isotope pattern of TTX ([M+H]^+^ C_11_H_18_N_3_O_8_, m/z 320.1088, Figure 4D) calculated using the SmartFormula software (Bruker Daltonics, Bremen, Germany). In addition, in the HR-LC-MS/MS spectrum (Figure 5), the major product ions detected at m/z 162.0681 and 302.0957 from the precursor ion of TTX [M+H]^+^ m/z 320.0863 detected in the scallops collected on August 22 (A) were close to those obtained from the authentic TTX (m/z 162.0637 and 302.0939) (B). The possible structures of these product ions were reported previously [25]. These data confirmed the identification of TTX in the hepatopancreas of Japanese cultured scallops.

### 2.4. TTX Analogues in Scallops

**Figure 6:**
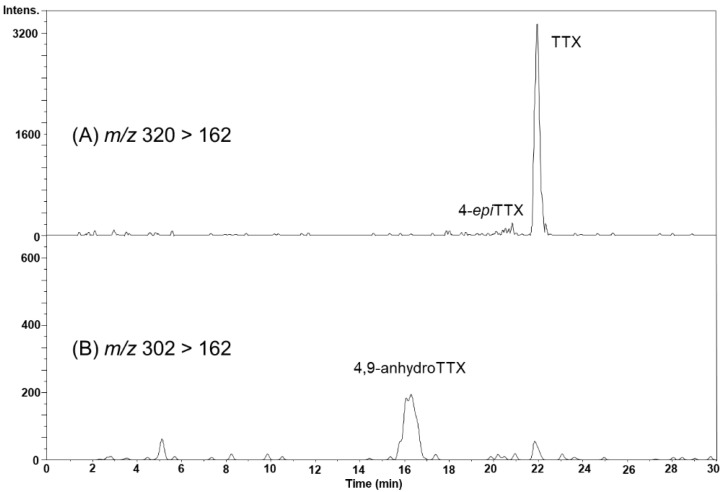
The LC-MS/MS (MRM) chromatograms of TTX and 4-*epi*TTX detected at *m*/*z* 320 > 162 (**A**), and 4,9-anhydroTTX in the hepatopancreas of scallops collected on August 22, 2017, detected at *m*/*z* 302 > 162 (**B**).

In the August 22 sample, the peak possibly corresponding to 4-*epi*TTX was detected on the MRM chromatogram at m/z 320 > 162 (Figure 6A), although this peak was not clearly detected by HR-LC-MS (Figure 4B). The peak of 4,9-anhydroTTX was detected on the MRM chromatogram at m/z 302 > 162 (Figure 6B). The concentrations of TTX and 4,9-anhydroTTX in the hepatopancreas were estimated to be 421 and 96.6 nmol/kg, respectively.

### 2.5. Temporal Variation of PSTs and TTX in Scallops, and the Cell Densities of Alexandrium tamarense and A. catenella

**Figure 7:**
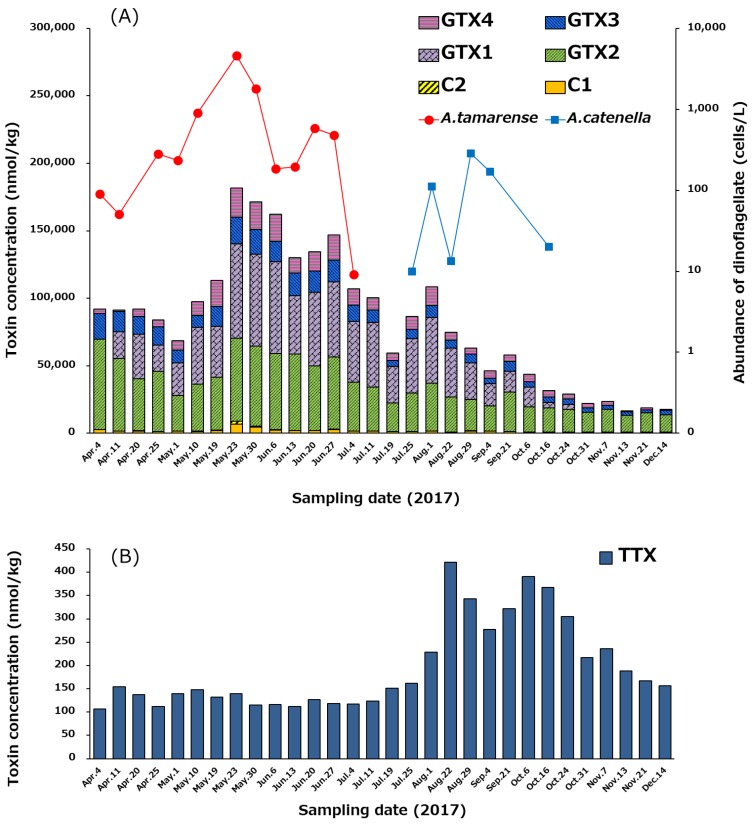
Temporal variation of the profile and concentrations of PSTs (**A**) and TTX (**B**) in the hepatopancreas of the scallops collected from April 4 to December 14 in 2017, and changes of the cell densities of *Alexandrium tamarense* and *Alexandrium catenella* in the area where the scallops were collected (**A**). The cell densities higher than 10 cells/L were plotted. The concentration of toxins was determined using LC-MS/MS.

The temporal variation of the profile and concentrations of PSTs and TTX in the hepatopancreas of scallops from April 4 to December 14, 2017, is shown in Figure 7. At the beginning of April, total PSTs level was 92,084 nmol/kg, and reached its highest value (181,748 nmol/kg) on May 23 (Figure 7A). Subsequently, the concentration of PSTs gradually decreased until December 14 (17,740 nmol/kg). GTXs complied more than 94% (mol/mol) of the PSTs detected in almost all periods examined, whereas C1/C2 were minor components. The congeners that had 11-α-OSO_3_H (C1, GTX1, GTX2) were dominant compared to 11-β-OSO_3_H congeners (C2, GTX3, GTX4) in all periods. At the beginning of April, GTX2 was the major component, but subsequently, GTX1 (N1-OH toxin) significantly increased, and the ratio of GTX1 to GTX2 was kept approximately 1:1 (mol/mol) until October 6. Then, GTX1 gradually decreased, while GTX2 did not decrease significantly until December 14. GTX5 was detected in almost all samples, but their concentrations were below the LOQ (<244 µg/kg).

As shown in Figure 7B, the concentration of TTX was in the range of 100–150 nmol/kg in the hepatopancreas from April 4 to July 25, and then increased to its highest level (421 nmol/kg) on August 22. Thereafter, the TTX level was maintained at more than 300 nmol/kg until October 24, before gradually decreasing to 157 nmol/kg on December 14. The trend of the temporal variation of concentration of TTX was clearly different from that of PSTs (Figure 7A).

As the major PSTs producing dinoflagellates in the area where the scallops were collected, *Alexandrium tamarense* flourished between January 5 (not shown in Figure 7A until April 4) and July 4 in 2017 (>10 cells/L), and then *Alexandrium catenella* bloomed from July 25 to October 16 (>10 cells/L). The maximum cell densities of *A. tamarense* (4625 cells/L) and *A. catenella* (284 cells/L) were counted on May 23 and August 29, respectively, as shown in Figure 7A. Total PSTs in the hepatopancreas reached the highest level on the day (May 23), when the cell density of *A. tamarense* peaked. Linkage of PSTs level to the cell density of *A. tamarense* was suggested as reported previously [60].

### 2.6. Concentration of PSTs and TTX in Whole Edible Tissues of Scallops

**Figure 8:**
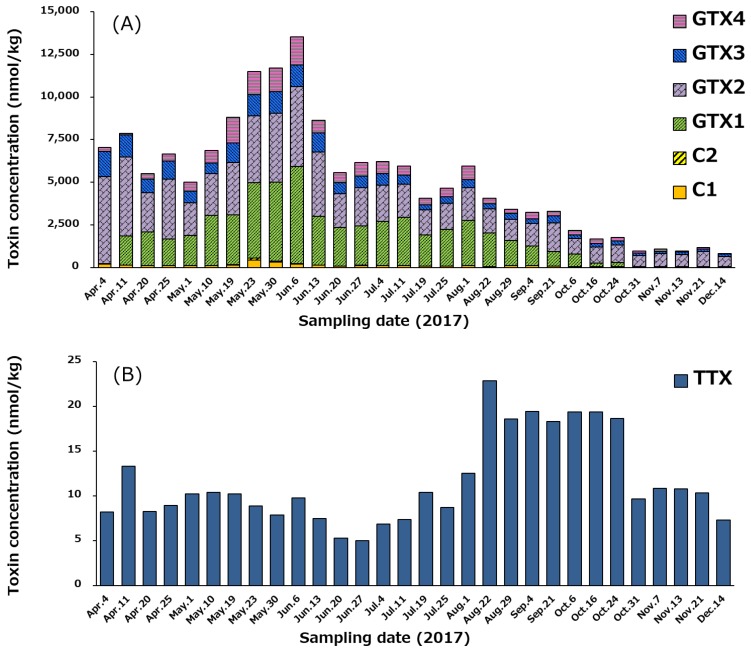
Temporal variation of the profile and concentrations of PSTs (**A**) and TTX (**B**) in the whole edible tissue including the hepatopancreas of the scallops collected from April 4 to December 14 in 2017. See Section 4.6. for an estimation of toxin concentration.

The temporal variation of the profile and concentrations of PSTs and TTX in whole edible tissues of scallops from April 4 to December 14, 2017, is examined and the results are shown in Figure 8, because whole edible tissues of scallops including hepatopancreas are sometimes consumed in Japan. A similar trend of the temporal variation to that of hepatopancreas is shown in Figure 7. Even though STX and neoSTX were excluded because of regulation (see Section 2.2), it should be noted concentration of PSTs in the whole edible tissue including the hepatopancreas was higher than the Codex threshold of 800 µg STX eq./kg [59] from April 4 to September 4 (Figure 8A); the concentration peaked on June 6 (3866 µg STX equivalents/kg, 13,547 nmol/kg). The maximum concentration of TTX (23 nmol/kg) in the whole edible tissue including the hepatopancreas occurred on August 22 (Figure 8B). This was lower than the level that EFSA proposed not to result in adverse effects in humans (138 nmol TTX equivalents/kg = 44 µg TTX equiv./kg shellfish meat) [39]. All scallop samples tested in this study cleared this food sanitation standard for TTX.

## 3. Discussion

In the present study, the temporal variation of the profile and concentrations of PSTs and TTX in scallops cultured in a bay of east Japan from April 4 to December 14, 2017 was examined. As mentioned in Section 2.5 for PSTs, at the beginning of April, GTX2 was the major component, but subsequently, GTX1 (N1-OH toxin) significantly increased, and the ratio of GTX1 to GTX2 was kept approximately 1:1 (mol/mol) for a while. Then, GTX1 gradually decreased, while GTX2 did not decrease significantly. It has been reported that 11-β-OSO_3_H toxins progressively transform into 11-α-OSO_3_H toxins, and N1-OH toxins (GTX1/4) are reduced to N1-H (GTX2/3) toxins in bivalves [60,61]. Our present results of the toxin profile in scallops (Figure 7A) are consistent with these reports. It is speculated that 11-β-OSO_3_H toxins (GTX3, GTX4) provided from dinoflagellates transformed into 11-α-OSO_3_H toxins (GTX1, GTX2) in scallops, and then, GTX1 was gradually released from scallops, but partially transformed to GTX2 by reduction of N1-OH to N1-H in the hepatopancreas of scallops. The order of epimerization at C11 and reduction of N1-OH is unclear, but it is predictable that C11 epimerization by keto-enol tautomerism would occur first, because the ratio of GTX1 drastically increased when total PSTs increased. This predicted metabolism in scallops could explain the reason why GTX2 was the major toxin after the level of toxins decreased to low levels (from October 16 to December 14).

In this study, STX and neoSTX were not determined because of Japan’s regulation for use of STX. In Japan, the mouse bioassay (MBA) for paralytic shellfish poisoning (PSP) toxins has been used in the official Japanese method [62], although alternative methods are being considered. We have preliminary tested the toxins from scallops in mice. As a result, the calculated toxicity based on relative toxicity reported by Oshima for PSTs [63] was almost coincidence to the toxicity in mice. This indicates that STX and neoSTX levels were not high to influence on the toxicity of scallops to mice in this study (see, Section 2.2). As described in Section 4.6, PSTs levels in the scallop tissue that did not include the hepatopancreas were below LOD for all samples used in this study. However, toxins levels in such tissue should be continuously monitored, because we have not confirmed other cases, for example, that the concentrations of PSTs in hepatopancreas are much higher than those measured in this study.

By comparing the concentrations of PSTs and TTX chronologically (Figure 7), it is clear that they do not follow the same trend. The concentration of PSTs in the scallops was the highest on May 23, which was linked to the cell density of *A. tamarense* (Figure 7A). In contrast, the concentration of TTX in the scallops was only 140 nmol/kg on May 23. TTX level in scallops was highest on August 22 (Figure 7B and Figure 8B), while at that time the density of *A. tamarense* was below 10 cells/L. This result suggests that the origin of TTX in scallops cannot be from *A. tamarense.* Turner et al. [32] reported that the concentration of TTX increases in bivalve shells around the United Kingdom during the summer. A similar pattern was observed in our data where TTX concentration in the scallops collected in a bay of east Japan peaked around summer (August 22) as described above.

Kodama et al. [44,45] previously identified TTX in *A. tamarense*, and proposed that *A. tamarense* is the origin of TTX in the highly toxic scallop, *P. yessoensis*, during a bloom of *A. tamarense* in Japan. They reported that the maximum concentration of TTX and PSTs in the scallops cultured in Japan coincided with an *A. tamarense* bloom. The present study contradicts these results. In our case, the peak of TTX concentration in the scallops (August 22) was close to the peak of *A. catenella* cell density (August 29). However, we have not obtained any evidence relating *A. catenella* to TTX production. We believe that there are TTX-producing microorganisms other than *A. tamarense* in the water column, giving rise to TTX in the cultured scallops hung in the ocean at 12–16 m from the bottom. These microorganisms might be planktons or benthos originating from the bottom of the ocean and stirred up from conditions occurring during this time frame.

The concentrations of TTX in scallops were much lower than those in pufferfish, starfish, snails, crabs etc., collected in Japan [15,18,20,21,25]. One assumption for this reason may be the difference in the ability among these organisms to accumulate toxins. Alternatively, while pufferfish and others can ingest concentrated TTX through the food chain, scallops may directly obtained TTX from TTX-producing microorganisms, which may produce only trace amounts of TTX.

This study confirmed that TTX is present at low concentrations in Japanese scallops, similar to those in other areas in the world. However, the concentrations were below the level that EFSA proposed did not cause adverse effects in humans (138 nmol TTX equivalents/kg = 44 µg TTX equivalents/kg shellfish meat). In the future, continuous monitoring of PSTs and TTX concentrations in scallops in the same region should be implemented. In addition, microorganisms, bacteria and planktons, upon which the scallops feed should be collected and tested. This will allow for the collection of the information necessary to provide evidence of the origin of TTX in scallops.

## 4. Materials and Methods

### 4.1. Reagents and Chemicals

The reagents for sample preparation and SPE (solid phase extraction) purification were HPLC grade and purchased from Wako Pure Chemical Industries (Osaka, Japan). HPLC or LC-MS grade acetonitrile (CH_3_CN), hydrochloric acid, formic acid, ammonium acetate (salt) and n-hexane were purchased from Kanto Chemical (Tokyo, Japan). Ammonium formate (salt) for LC-MS was purchased from Sigma-Aldrich (St. Louis, MO, USA). The standards for GTX1 and 4, GTX2 and 3, GTX5 and 6, decarbamoyl GTX2 and 3 (dcGTX2 and 3), C1 and C2, and dcSTX were purchased from the National Research Council of Canada (NRCC, Halifax, Nova Scotia, Canada). TTX was purchased from Wako Pure Chemical Industries. This TTX contained a small amount of 4,9-anhydroTTX that was used as a standard of 4,9-anhydroTTX. This 4,9-anhydroTTX was identified using the standard of TTX analogues previously prepared from pufferfish [24,25]. These standards were diluted with CH_3_CN or 0.1% acetic acid (v/v) as needed. Dilution of the standards solution, originally in diluted acetic acid, with CH_3_CN effectively prevented peak broadening. The solubility of toxins in CH_3_CN containing slight amounts of diluted acetic acid was confirmed.

### 4.2. Validation of the HILIC-MS/MS Method

According to the Codex guideline [7] method validation for range of calibration curves, LOD and LOQ for standards (Table 1) were confirmed using the Analyst software (ABSciex) for each PST toxin. The concentration range of the calibration curves had been set for each standard poison component: C1 (44–703 nmol/L, *r* = 0.9997), C2 (13–2104 nmol/L, *r* = 0.9650), GTX1 (771–6174 nmol/L, *r* = 0.9991), GTX2 (334–2656 nmol/L, *r* = 0.9872), GTX3 (32–1012 nmol/L, *r* = 0.9698), GTX4 (30–972 nmol/L, *r* = 0.9901), GTX5 (17–66 nmol/L, *r* = 0.9579), GTX6 (71–283 nmol/L, *r* = 0.9791) and TTX (4–31 nmol/L, *r* = 0.9766) (5 µL injection). 4,9-anhydroTTX was quantified using a calibration curve for TTX. For LOD and LOQ of sample, 1 mL of the sample solution was prepared from 0.5 g scallop tissue as described in Section 4.5. The recovery of toxins through sample preparation procedure was approximately 80% for all toxins. This sample solution was diluted five or 10 times, after which 5 µL was injected. Therefore, the LOD and LOQ for samples (nmol/kg) were calculated as 12.5 times (in the case of five times dilution) the value of the LOD (nmol/L) and LOQ (nmol/L) for the standard, respectively, without consideration of matrix effects.

### 4.3. Scallop Samples

The scallops analyzed in this study were chronologically collected in a bay of east Japan at the same geographical area from April 4 to December 14, 2017. Two year cultured scallops were purchased and hung in the ocean at 12–16 m from the bottom, and 8–12 m from the surface of the sea, at the beginning of January of 2017 for the present experiments. Five shellfish specimens were collected each week. The size of each specimen was approximately in the range from 10 × 10 cm to 15 × 15 cm, and the weight of each specimen was in the range from 173 g to 489 g. A description of the exact area from where the scallop samples were collected was avoided to prevent damaging the reputation of the scallop producers in the region. The scallops were frozen and stored at −30 °C until use.

### 4.4. Cell counts of A. tamarense and A. catenella

For enumeration of *A. tamarense* and *A. catenella* vegetative cells, 500 mL aliquots of each seawater sample were collected from the positions of each meter between 0 and 23 m from the surface of the ocean in 2017 including the term from April 4 to December 14. Each seawater sample was concentrated to 5 mL with plankton net (net mesh size 20 µm). One time count was made for 1 mL aliquots of the concentrated samples under a light microscope. The detection limit of this procedure is 10 cells/L. The average of the counts of each depth denoted the count.

### 4.5. Toxin Extraction and Clean-Up

Toxins in the hepatopancreas (digestive gland) of scallops were extracted according to the AOAC method [64] and previous studies [56]. The hepatopancreas from five specimens was excised and homogenized by a blender. The hepatopancreas homogenate (3.0 g) was mixed with 0.1 M HCl (3 mL) and boiled for 5 min. After cooling to room temperature, the crude extract was mixed with hexane (3 mL) and centrifuged at 9530 g for 5 min to remove the hexane layer. After repeating this defatting procedure twice, the water layer was passed through an OASIS-HLB cartridge (60 mg/3 mL, Waters, Milford, USA), which was conditioned with methanol (2 mL). The passed solution was filtered through an ultra-filter (10,000 MW cut-off, Amicon-Ultra 4, Merck Millipore, Burlington, USA) by centrifugation at 7500 g for 20 min to obtain the toxin extract solution. Next, the solution was diluted five or 10 times with CH_3_CN before LC-MS/MS analysis. The toxin extract solution for HR-LC-MS analysis was further purified by passing the solution through a Supelclean ENVI-Carb PSA/SPE (500 mg/500 mg, 6 mL, Merck, Darmstadt, Germany) according to the method previously described [50]. Sample solutions from whole edible tissues except hepatopancreas were prepared using the same method used for the hepatopancreas as described above.

### 4.6. Estimation of Toxin Concentration in the Whole Edible Tissue

Toxin levels in tissue that did not include the hepatopancreas were below LOD for all samples. In order to estimate the total toxin concentration in the edible tissue of our samples we used the toxin levels present in the hepatopancreas as our measure of total toxin (data from Figure 7). These values were then divided by the total whole weight of edible tissue (including the hepatopancreas) to provide an estimate of edible tissue concentrations for our samples.

### 4.7. LC-MS/MS for Simultaneous Analysis of PSTs and TTXs in Scallops

LC-MS/MS in MRM mode was performed on an API-4000 triple quadrupole mass spectrometer (ABSciex, Framingham, USA) coupled to an Agilent 1100 HPLC (Santa Clara, USA). The HPLC system included a solvent reservoir, a degasser (G1379A), a binary pump (G1312A), a refrigerated autosampler (G1367A) and a column oven (G1316A). The LC conditions for simultaneous analysis of PSTs and TTXs in this study were set up by modification of the method reported by Thomas et al. [52]. In our conditions, a TSK-gel Amide-80 column (2 µm, 2.0 mm × 150 mm, Tosoh Bioscience, Tokyo, Japan) was used instead of a TSK-gel Amide-80 column (5 µm, 2.0 mm × 250 mm) [52] to reduce the analytical time, and ammonium acetate in the solvent was used instead of ammonium formate [52] to improve the LOD, LOQ values of standard toxins using our MS spectrometer. The toxins were separated on the above described column at 40 °C. The mobile phase consisted of (A) 10 mM ammonium acetate (salt) with 0.05% formic acid solution and (B) CH_3_CN, and the flow rate was 0.2 mL/min. The elution program was applied as follows: 75% B isocratic to 11 min, gradient elution from 75% B to 45% B over 9 min, hold 45% B for 4 min, gradient elution from 45% B to 35% over 2 min, hold 35% B for 3 min, gradient elution from 35% B to 75% B over 1 min, hold 75% B for 10 min (post run). The injection volume was 5.0 µL. For HR-LC-MS analysis, ammonium acetate in the above solvent was replaced by ammonium formate. The ionization parameter settings were as follows: collision gas (CAD), 9 psi; curtain gas (CUR), 30 psi; nebulizer gas pressure, 60 psi; auxiliary gas, 40 psi; spray voltage, 5500 V; probe gas temperature, 500 °C. The MS/MS parameters for the 12 toxins in MRM mode (Table 1) were carried out in the positive ionization mode.

### 4.8. HR-MS/MS Method

HR-LC-MS/MS analysis for TTXs [25] was performed on a MicrOTOFQII (Bruker Daltonics, Bremen, Germany) coupled to a Nexera UHPLC system (Shimadzu, Kyoto, Japan). The separation column and gradient conditions were the same as those for the LC-MS/MS measurement described above, although ammonium acetate in the mobile phase for LC-MS/MS was replaced by ammonium formate in the mobile phase for HR-LC-MS/MS. The conditions of the MS spectrometer were as follows: positive ionization mode, dry gas 7 L/min, dry temperature 180 °C, nebulizer 1.6 Bar, capillary 4500 V. MS/MS was performed in MRM mode setting [M+H]^+^ as the precursor ions. The precursor ions and sweeping collision energy was 320.1088 ± 0.05, 36.9–55.4 eV for TTX.

## 5. Conclusions

The profile and concentrations of PSTs and TTX in the hepatopancreas of scallops cultured in a bay of east Japan, collected from April to December 2017, were analyzed. The highest concentration of PSTs was detected on May 23, whereas the highest TTX concentration was detected on August 22. Contrary to the previous report, the temporal changes in PSTs and TTX concentrations did not overlap. TTX levels in the scallops were much lower than that of PSTs in all samples tested. The highest concentration of TTX in the whole edible tissue was 23 nmol/kg (7.3 µg/kg), recorded on August 22, which is well below the level that EFSA proposed not to result in adverse effects in humans (138 nmol TTX equivalents/kg, 44 µg TTX equivalents/kg shellfish meat). Further in-depth study is needed to understand the origin of TTX in cultured scallops.

## Figures and Tables

**Table 1:** MRM conditions and LOD and LOQ for PSTs and TTX analogues

Analyte	Precursor Ion	Product Ion	DP (V)	CE (V)	Standard LOD	Standard LOQ	Sample LOD	Sample LOQ
	(*m*/*z*)	(*m*/*z*)			(nmol/L)	(nmol/L)	(nmol/kg)	(nmol/kg)
C1	396	316	46	17	8	17	105	210
C2	396	298	66	23	6	11	79	131
GTX1	412	314	65	19	221	440	2765	5500
	412	332	65	19	255	510	3190	6381
GTX2	396	316	46	17	167	334	2086	4173
GTX3	396	298	56	23	13	25	158	316
	396	316	56	17	25	51	316	632
GTX4	412	394	65	17	24	49	304	608
	412	314	65	23	17	24	213	304
GTX5	380	300	46	19	11	20	132	244
	380	204	46	39	29	58	362	725
GTX6	396	316	46	19	33	96	411	1201
	396	298	46	39	91	182	1138	2276
dcGTX2	353	273	41	19	99	199	1242	2484
dcGTX3	353	335	41	17	17	37	213	461
dcSTX	257	239	85	21	252	507	3152	6341
	257	180	95	29	109	122	1367	1519
TTX, 4-*epi*TTX	320	162	80	51	1	2	16	30
4,9-anhydroTTX	302	162	80	41	-	-	-	-

DP declustering potential, CE collision energy, LOD *s*/*n* = 5, LOQ *s*/*n* = 10. Injection volume was 5 µL. Samples are hepatopancreas of scallops.

## Supplementary Materials

The following are available online at https://www.mdpi.com/1660-3397/17/12/653/s1, Figure S1: HR-MS spectrum of 12β-deoxyGTX3 in the hepatopancreas of scallops, Figure S2: HR-LC-MS chromatograms and HR-LC-MS/MS spectra of authentic 12β-deoxyGTX3 and the sample solution from the hepatopancreas of scallops, Figure S3: HR-LC-MS chromatograms of authentic 12β-deoxyGTX3, 12β-deoxyGTX2/3, and the sample solution under column switching condition.
