# Supplementary material for: Temporal Variation of the Profile and Concentrations of Paralytic Shellfish Toxins and Tetrodotoxin in the Scallop, Patinopecten yessoensis, Cultured in a Bay of East Japan
Source: Mar Drugs. 2019 Nov 21;17(12):653. doi: 10.3390/md17120653 (PMC6950525; doi:10.3390/md17120653)
Supplement: Supplementary file 1 [file marinedrugs-17-00653-s001.pdf]

Supporting Information

Temporal variation of the profile and concentrations of paralytic shellfish toxins and tetrodotoxin in the scallop, *Patinopecten yessoensis,* cultured in a bay of east Japan

Satoshi Numano, Yuta Kudo, Yuko Cho, Keiichi Konoki and Mari Yotsu-Yamashita*

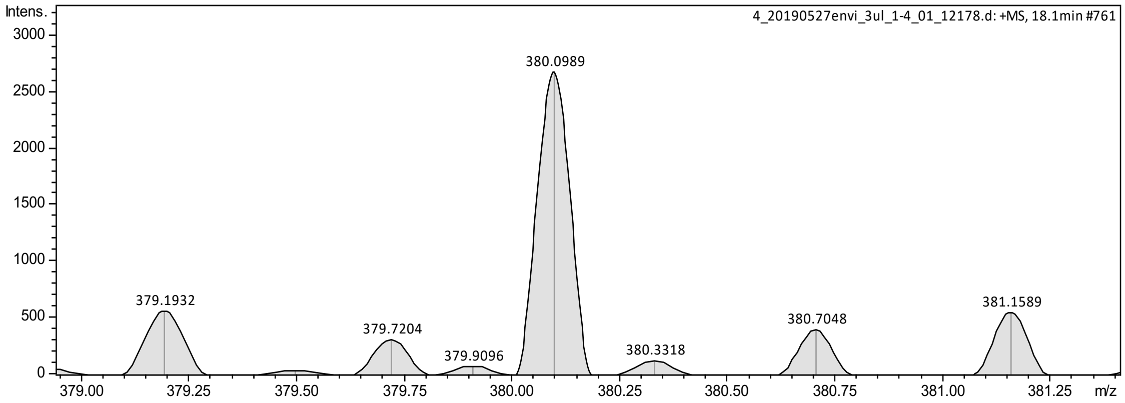

Figure S1. HR-MS spectrum of 12β-deoxyGTX3 detected in the hepatopancreas of scallops.

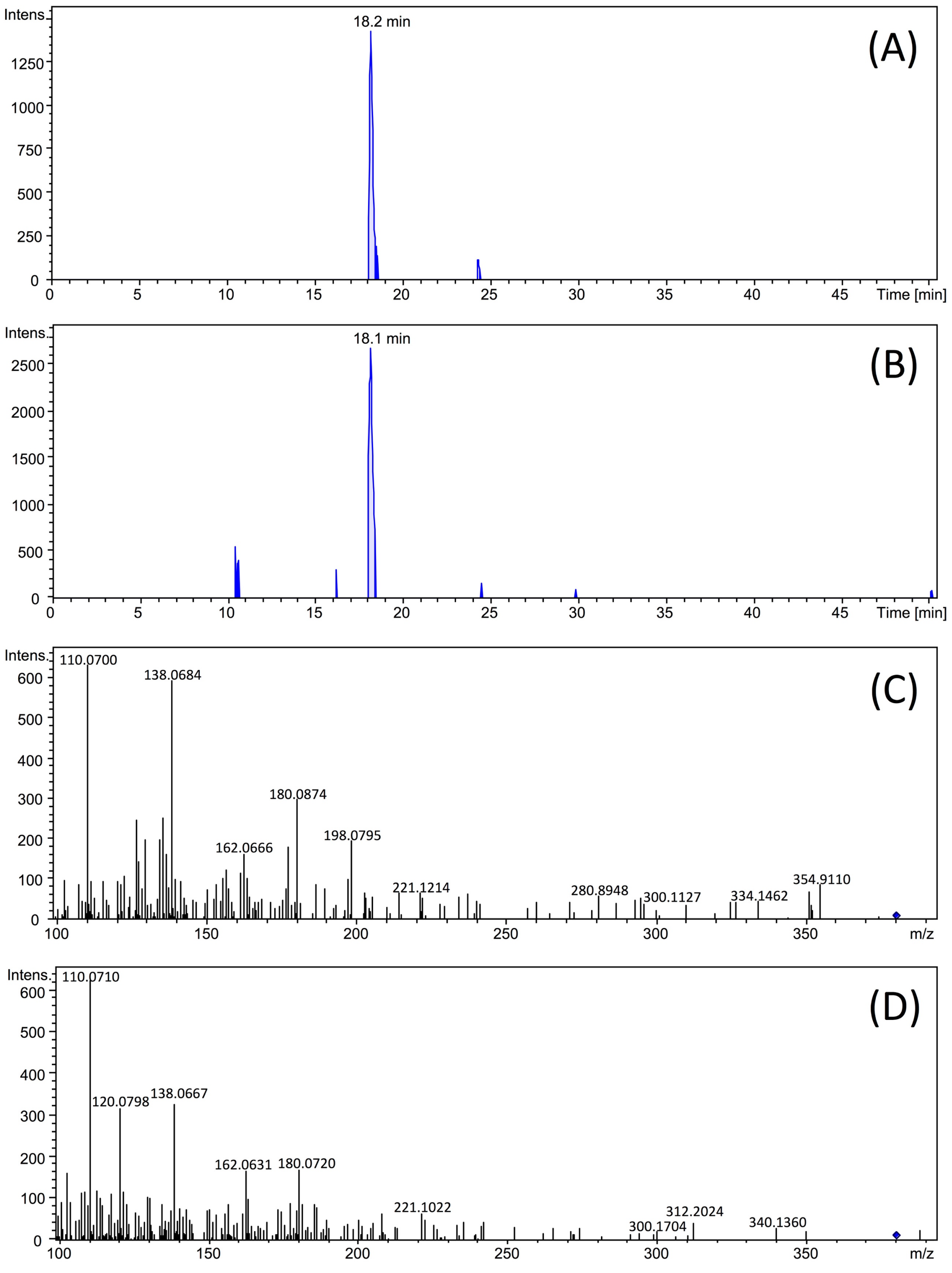

Figure S2. HR-LC-MS chromatograms (A, B) detected at *m/z* 380.0979 ± 0.05 and HR-LC-MS/MS spectra (C, D) of authentic 12β-deoxyGTX3 (A, C) and the sample solution from the hepatopancreas of scallops (B, D). The retention time of 12β-deoxyGTX3 was

different from those in Figures 2, 3, and 5, using the slightly different solvent for LC-MS from that for those figures (see, Section 4.7). The HR-LC-MS conditions are same as those described in text (Section 4.8) without MS/MS.

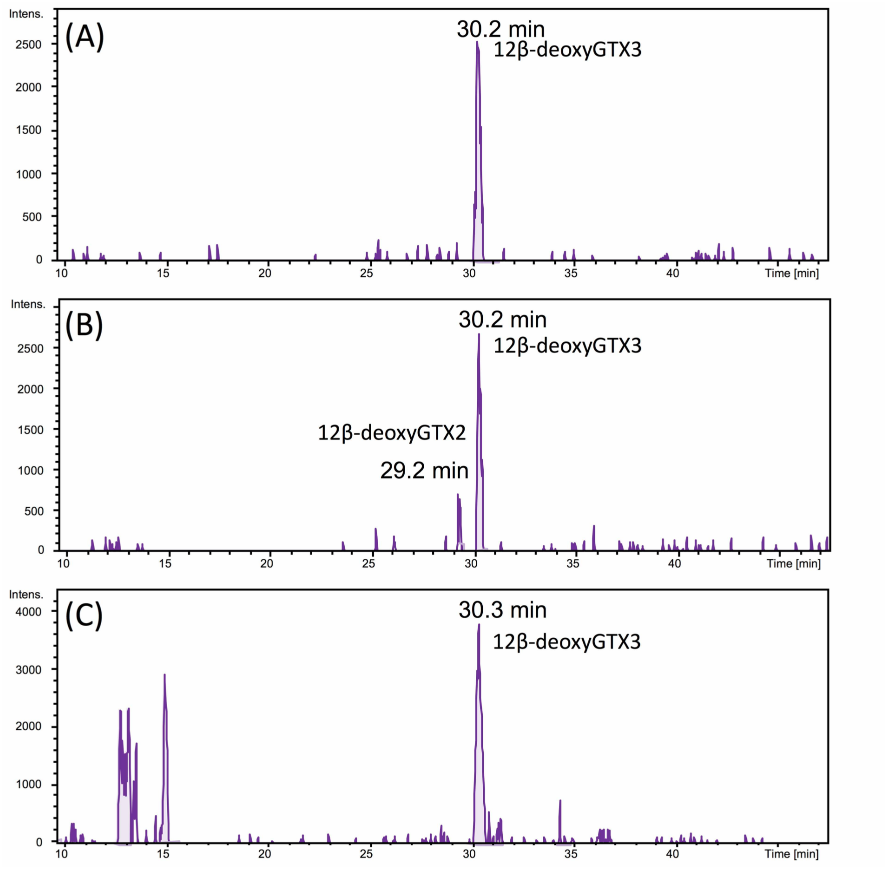

Figure S3. HR-LC-MS chromatograms of synthetic 12β-deoxyGTX3 (A) [53], the mixture of synthetic 12β-deoxyGTX2 and 12β-deoxyGTX3 (B), and the sample solution from the hepatopancreas of scallops (C) detected at *m/z* 380.0979 ± 0.05 under the column switching condition reported in [54].
